# Use of Fatigue Index as a Measure of Local Muscle Fatigability in Ryanodine Receptor Isoform-1-Related Myopathies

**DOI:** 10.3389/fneur.2019.01234

**Published:** 2019-12-10

**Authors:** Jessica W. Witherspoon, Julie S. Rekant, Paul G. Wakim, Ruhi Vasavada, Melissa Waite, Irene Chrismer, Monique O. Shelton, Minal S. Jain, Katherine G. Meilleur

**Affiliations:** ^1^Neuromuscular Symptoms Unit, National Institute of Nursing Research, National Institutes of Health, Bethesda, MD, United States; ^2^Rehabilitation Medicine Department, Mark O. Hatfield Clinical Research Center, National Institutes of Health, Bethesda, MD, United States; ^3^National Institutes of Health Clinical Center, Biostatistics and Clinical Epidemiology Service, Bethesda, MD, United States

**Keywords:** fatigue index, *RYR1*-RM, fatigability, muscle, neuromuscular

## Abstract

**Introduction:** Individuals affected with ryanodine receptor isoform-1-related myopathies (*RYR1-*RM) commonly experience fatigability in the quadriceps, which may limit physical function and potentially diminish quality of life. Fatigability, in *RYR1*-RM, results from skeletal muscle injury secondary to dysfunction of the major skeletal muscle Ca^++^ channel. However, during fatigability testing, affected individuals did not always reach the point of local muscle fatigue as defined by a fatigue index (FATI) at 50% of peak torque. Surakka et al. compared three versions of FATI equations, which vary by the area under the force curve (AUC). By performing this comparison, they were able to determine the optimal equation in individuals with Multiple Sclerosis.

**Purpose:** Using a similar comparison, we sought to identify the optimal FATI equation in the *RYR1*-RM population. Secondly, because local muscle fatigability might have an impact on independent living, this study also assessed change in local muscle fatigability over a 6-month time frame.

**Methods:** Thirty participants were analyzed from the *RYR1*-RM natural history study and double-blind, placebo-controlled N-acetylcysteine (NAC) trial, NCT02362425. Twenty-seven had fatigability data, from isometric knee extension and flexion fatigability tests, available for the purpose of establishing a method for predicting FATI at 50% peak torque. For the natural history study, 30 participants were used to assess disease progression of local muscle fatigability achieved during the knee extension fatigability test, and 29 participants for the knee flexion fatigability test.

**Results:** Surakka's equation 1, using the prediction approach, led to the smallest median error, the smallest square-root of uncorrected sum of squares, and the smallest average of the absolute value of the differences. No difference was observed in FATI at 50% peak torque between month 0 and month 6 for extension (*p* = 0.606) and flexion (*p* = 0.740).

**Conclusion:** Surakka's equation 1, with the prediction approach, was found to be the most accurate for imputing values when fatigue was not reached during a sustained knee isometric fatigability test in *RYR1*-RM. Furthermore, when used to assess fatigability-based disease stability, local muscle fatigability, in this RYR1-RM population remained stable.

## Introduction

Fatigue and muscle weakness are the two most commonly reported symptoms in individuals with ryanodine receptor isoform-1-related myopathies (*RYR1-*RM). Depending on disease severity, these symptoms may limit physical function and potentially diminish quality of life (1). Individuals affected with *RYR1-*RM experience primary fatigue (2) at the level of the peripheral muscles (3). More specifically, calcium (Ca^++^) dysregulation occurs because of dysfunction of the major skeletal muscle Ca^++^ channel, RyR1, which results in impaired excitation-contraction coupling (ECC), excessive mitochondrial oxidative stress, and hyperactive post-translational modifications that contribute to skeletal muscle injury (4). An optimal means for quantifying fatigability, defined in the population of neuromuscular disease as the reduction in one's ability to generate force or output power (5), has yet to be determined in *RYR1-*RM.

The quantification of fatigability in ambulatory and non-ambulatory individuals with *RYR1-*RM has the potential to facilitate future clinical trials by serving as an outcome measure in the determination of therapeutic efficacy. Historically, primary vs. secondary fatigue and central vs. peripheral fatigue have been terms used to define qualitative (subjective) and quantitative (objective) fatigue (2). Recently, the construct of fatigue was limited to subjective measures, whereas fatigability was defined as a construct that is measured objectively (2). Likewise, here, the term fatigue refers to subjective sensations, whereas fatigability is defined as objective changes in performance (2, 6). Although there are several affected skeletal muscles in *RYR1-*RM, the quadriceps is one of the most commonly affected muscle groups, impairing individuals' functional abilities such as stair climbing, transferring positions, and running and/or prolonged walking (7, 8). Our previous study showed that the 6-min walk test (6MWT) can be used to measure general fatigability in ambulatory individuals affected with *RYR1*-RM (9). To complement our previous work, the present study aimed to identify an outcome measure capable of quantifying local muscle fatigability (10). Fatigability, in local muscle groups, is quantified using fatigue indices (FATI) (3) during repetitive or sustained muscle contractions. However, a variety of methods exist to calculate FATI. FATI, first described by Djaldetti et al. (11) and later by Surakka et al. (12), have been used to quantify declines in force production during sustained muscle contractions in individuals with Multiple Sclerosis. A FATI at 50% of peak torque has also been used to define the point of fatigue (13, 14). Surakka et al. (12) compared three versions of FATI equations, which vary by the area under the force curve (AUC). By performing this comparison, they were able to determine the optimal equation in individuals with Multiple Sclerosis (3, 12).

Using a similar comparison, we sought to identify the optimal FATI equation in our population of interest, *RYR1*-RM. The differences in the AUC used for each of the three FATI equations above are based on the peak used from the fatigability test. In equation 2 (Supplementary Figure 2), peak torque is the torque achieved at the 5 s mark, while equations 1 (Supplementary Figure 1) and 3 (Supplementary Figure 3) incorporate the highest torque within the first 5 s. However, equations 2 and 3 only include the portion of the test where the subject's force production is declining, whereas equation 1 also accounts for the portion of the test where the participant's force is increasing toward its peak. Overall, Equation 1 incorporates the entire fatigability test, while the other two equations are limited to the portion of the test that occurs after the subject has produced maximum torque. In the *RYR1*-RM population, we found that, during fatigability testing, affected individuals did not always reach 50% of their peak torque in a 30- or even 60-s trial. The first goal of this study was to determine which FATI equation best predicts fatigability at 50% peak torque based on the FATI at 30 s during a sustained contraction knee fatigability test.

Secondly, because local muscle fatigability might have an impact on independent living, this study also assessed change in local muscle fatigability over a 6-month time frame. *RYR1-*RM have been clinically identified as stable or slowly progressive diseases, which we recently substantiated within this time frame (9, 15, 16). Therefore, after determining the optimal equation in this population, it was then used to impute the FATI at 50% peak torque for participants who did not achieve 50% peak torque in 30 s. This allowed us to observe whether fatigability was stable over 6 months in this population.

## Methods

### Fatigability Testing

#### Participants

**Figure 1:**
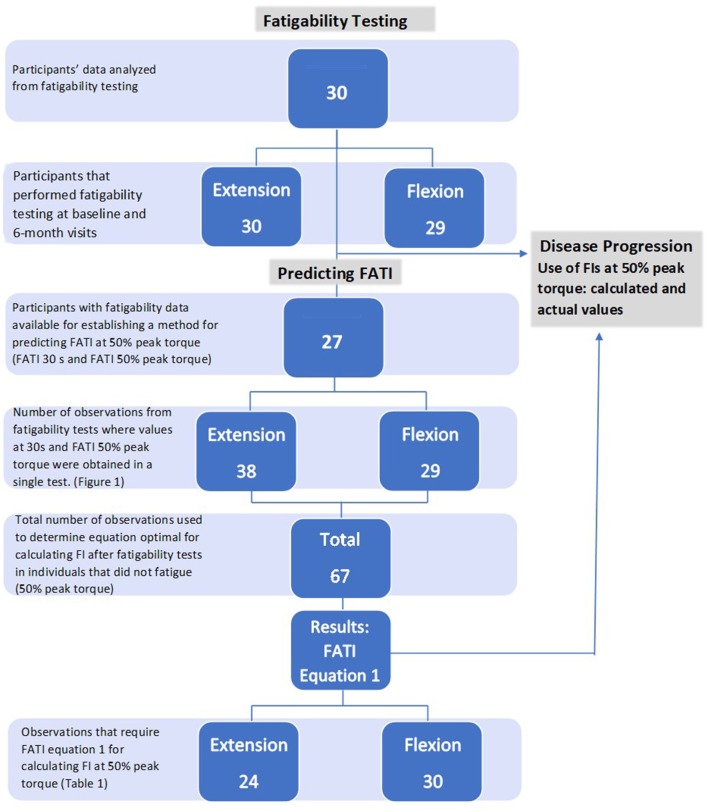
Diagram of participants and observations used for analysis. Natural history (section Fatigability Testing); predicting FATI (section Prediction Method for FATI); disease progression (section Disease Progression).

A total of 30 participants' data were analyzed from the *RYR1-*RM natural history study and double-blind, placebo-controlled N-acetylcysteine (NAC) trial (Table 1, Figure 1). The National Institutes of Health (NIH) Combined Neurosciences Institutional Review Board approved the trial (NCT02362425). Adult participants provided written informed consent. Written consent for minors was obtained from parents/caregivers with written assent from the child (<18 years). Inclusion criteria were: >7 years of age, ambulatory, clinical symptoms of fatigue and weakness consistent with *RYR1*-RM, and a confirmed genetic diagnosis of *RYR1-*RM. Participants had diagnoses of Central Core Disease, Multi-mini Core Disease, and Centronuclear Myopathy, many with malignant hyperthermia susceptibility. Participants were excluded if they had a history of liver or lung disease, ulcers, or dysphagia, were pregnant or breastfeeding, or had plans to become pregnant. To avoid potential strain on the knee from fatigability testing, individuals with a history of knee injury were excluded from isokinetic strength testing. Participants were seen at baseline and pre-treatment (6 months) for the natural history study.

**Table 1:** Demographic information for participants used in this study.

**Demographics**	***n* = 30**
^a^Age at evaluation, years	26 ± 17.6
^a^BMI, kg/m^2^	22.0 ± 7.6
^b^Gender, ♂:♀	13:17
^b^Mode of Inheritance (AD/DN:AR)	20:10

a *Data are expressed as mean ± standard deviation*.

b *Data are expressed as frequency*.

*AD/DN, Autosomal Dominant/De Novo; AR, Autosomal Recessive*.

#### Measurements

**Figure 2:**
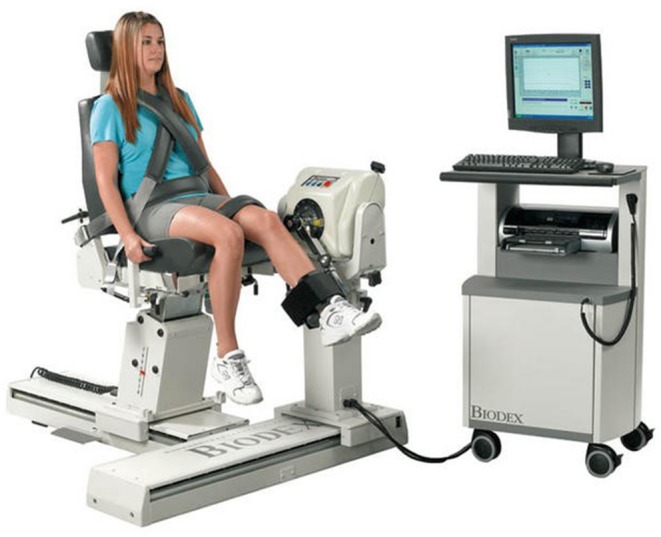
Testing set (Biodex 3 Medical Systems Inc., Shirley, NY, USA).

A dynamometer (Biodex 3 Medical Systems Inc., Shirley, NY, USA) was used for maximal isometric strength testing (Max Test) and fatigability testing (Fatigue Test) of the dominant lower limb. Participants were seated with hips in 85° flexion with the axis of the dynamometer shaft aligned with the anatomical rotational axis of the knee (Figure 2). The leg attachment was positioned slightly above the Achilles tendon, and range of motion parameters were established. Stabilization straps for the trunk, pelvis and thigh were used during testing to isolate knee extension and flexion, minimizing compensatory movements. All parameters for seat and dynamometer positions were documented to recreate for subsequent visits.

#### Testing

1. Max Test
  Prior to the start of test, the lower limb was positioned in 60° knee flexion. Standardized instructions were read to the participant to either kick out or pull back as hard and as long as possible. Two trials each for alternating maximal isometric knee extension and knee flexion were completed, with each trial lasting for 5 s with a 2-min reset between each trial.
2. Fatigue Test
  Participants remained seated in the same position as above for the fatigue test. The highest peak force achieved from the max strength test trials (extension and flexion) was used as a target for the corresponding fatigue tests. Standardized instructions were read to the participant to try to reach and maintain the peak force target threshold established during the max test. One trial of a 60 s knee extension fatigue test was followed by a 60 s knee flexion fatigue test, with a 2-min rest period between. If participants fatigued prior to the end of the 60 s test, they were instructed to remain still until the test period was finished. The participants were blinded to the length of the test before the start. After completion of the fatigability test, the participant rested for a minimum 5-min recovery period.

### Prediction Method for FATI

#### Participants

For this natural history study, 27 participants had fatigability data with 30 s FATIs available for the purpose of establishing a method to retrospectively predict FATI at 50% peak torque (Figure 1). Fatigability testing was performed during the trial to determine fatigue-related disease progression. In order to do this, a method for predicting FATI at 50% peak torque first needed to be established (b). The method required the use of cases with non-missing FATI at 30 s and non-missing FATI at 50% peak torque because the former was used to estimate the latter, and the value of the latter was used as the “true” value to calculate the estimation error. Based on these cases, there were 38 observations used from the extension fatigability tests and 29 observations used from the flexion fatigability tests for a total of 67 observations used in the prediction method (Figure 1). It is important to note that one, two, or three observations may be obtained from tests of a single participant, but at different time points.

#### Fatigue Indices

Data were down-sampled to 1 Hz using MATLAB. FATI equation 1 (FATI_1_) incorporated the entire length of time, including both the rise and fall phase of the fatigability testing window. FATI_1_ was calculated as the AUC over the length of the trial as a proportion of the total plot area (Supplementary Figure 1). The FATI_1_ equation is expressed as [1–(AUC_0−t_/[F_max(0−5)_ * t])] * 100 where t is the length of the trial, AUC_0−t_ is the AUC from time 0 to the end of the trial, and F_max(0−5)_ is the maximum force attained during the first 5 s of the trial.

FATI equation 2 (FATI_2_) and FATI equation 3 (FATI_3_) accounted for only the phase of the trial where force production was declining. In FATI_2_, this included the trial after the first 5 s. FATI_2_ was calculated as the AUC from 5 s to the end of the trial as a proportion of the total plot area from 5 s to the end of the trial (Supplementary Figure 2). The FATI_2_ equation is expressed as [1–(AUC_5−t_/[F_5_ * (t − 5])] * 100 where AUC_5−t_ is the AUC for the trial excluding the first 5 s and F_5_ is the force value at the 5 s timepoint.

For FATI_3_, a timepoint maximum (tpm) variable was introduced. This was defined as the time (during the first 5 s of the trial) at which the maximum force was reached. For FATI_3_ fatigability was calculated from this tpm to the end of the trial. FATI_3_ was calculated as the AUC from tpm to the end of the trial as a proportion of the total plot area from tpm to the end of the trial (Supplementary Figure 3). The FATI_3_ equation is expressed as [1–(AUC_tpm−t_/[F_max_ * (t–tpm)])] * 100 where AUC_tpm−t_ is the AUC for the trial from tpm to the end and F_max_ is the maximum force value reached during the first 5 s of the trial.

To gauge the speed of fatigability and to compare across groups, a timepoint 50 (tp50) was calculated as the time at which the subject's force output was 50% of their maximum force for that trial. Each of the three equations was calculated for *t* = 30 s, *t* = 60 s, and *t* = tp50, where appropriate (Table 2).

**Table 2:** FATI calculations for different timepoints during fatigability test.

***N*** **(extension, flexion)**	**Condition**	**FATI_**1, 2, 3**_ at *t* = 30 s**	**FATI_**1, 2, 3**_ at *t* = tp50 (50% peak torque)**
(36, 37)	Fatigued fully before 30 s	Unable to calculate	Calculated
(38, 29)	Fatigued fully between 30 and 60 s	Calculated	Calculated
(24, 30)	Did not fatigue fully during the length of the 60 s trial	Calculated	Unable to calculate

*N is the number of observations for (1) extension and (2) flexion per condition. Some observations may be repeat cases (refer to statistical methods). Only those who fatigued fully between 30 and 60 s were used in the present analysis*.

#### Statistical Method

SAS version 9.4 (SAS Institute, Cary, North Carolina) was used to perform all statistical analyses. For observations with non-missing FATI at 30 s and non-missing FATI at 50% peak torque, FATI at 50% peak torque was estimated based on FATI at 30 s using two different approaches: (1) Using the FATI at 30 s value itself; and (2) Fitting a linear regression model with FATI at 50% peak torque as the dependent variable and FATI at 30 s as the independent variable, and using that model to predict/estimate FATI at 50% peak torque. In the second approach, the regression model was fitted on all observations except the one for which the FATI 50% peak torque was being predicted. This was repeated for each observation.

To compare the 3 equations, errors between estimated and actual values of FATI at 50% peak torque were examined, and descriptive statistics for these errors were calculated. For the first approach, errors equaled the value of FATI at 30 s (considered as the estimated value of FATI at 50% peak torque) minus the actual value of FATI at 50% peak torque. For the second approach, errors equaled the value of the predicted value of FATI at 50% peak torque based on the regression model minus the actual value of FATI at 50% peak torque.

Because this analysis examined pairs of measurements, FATI at 30 s and FATI at 50% peak torque from months 0, 6, and 12 were included in the analyses, regardless of whether they came from the same participant at different times. There were 38 such pairs of measures from the extension fatigability test, and 29 pairs of measures from the flexion fatigability test.

To test whether there could be a time effect on the difference between FATI at 30 s and FATI at 50% peak torque (Approach #1), and between the predicted and actual values of FATI at 50% peak torque (Approach #2), an ANOVA with a “month” effect was fitted on the difference.

### Disease Progression

#### Participants

Baseline and pre-treatment data from all 30 participants were used to assess disease progression of local muscle fatigability achieved during the knee extension fatigability test, and 29 participants for the knee flexion fatigability test (Figure 1). See Table 2.

#### Statistical Method

To assess disease progression, univariate analyses including confidence intervals and student *t*-tests were used to compare the difference in FATI at 50% peak torque, including actual and imputed values, between month 0 and month 6.

## Results

Table 2 shows which FATIs were obtained following a 60 s isometric knee fatigability test. Based on this table, not all participants achieved fatigue as determined by FATI at 50% peak torque. Individuals who did not fatigue obtained a FATI at 30 s only. However, those who fatigued between 30 and 60 s obtained both a FATI at 30 s and at 50% peak torque, and therefore their data were further analyzed.

There was no month effect on the magnitude of the errors from either approach. For both extension and flexion, and for both approaches, *p*-values for the month effect ranged between 0.10 and 0.76. Results for each approach are discussed below.

### Approach 1

**Figure 3:**
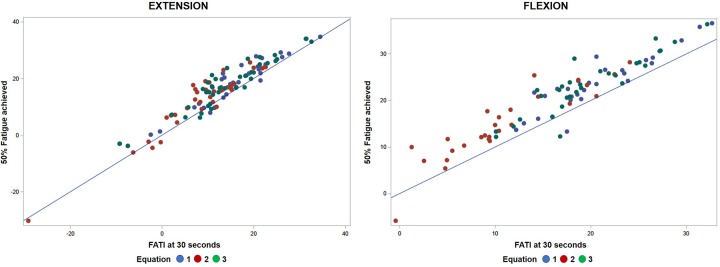
Comparative analyses of predicted FATI at 50% peak torque using Approach 1 (section Approach 1) and actual FATI at 50% peak torque for extension and flexion. Result: observations aggregate together relative to linear line.

The points in Figure 3 clustered above the 1:1 fit line shows FATI at 30 s tends to be slightly lower than its corresponding FATI at 50% peak torque. When comparing equations 1 through 3, equation 1 during extension (Table 3) leads to the smallest average difference (bias) (−2.46), the smallest standard deviation (spread of errors) (2.66), the smallest median difference (−2.35), the smallest square-root of uncorrected sum of squares (3.59), and the smallest average of the absolute value of the differences (2.90). A similar pattern is shown in Table 4 for flexion. In support, when observing the percent difference between the two values (FATI at 30 s minus FATI at 50% peak torque) over the actual magnitude of the measures, FATI at 30 s tends to underestimate FATI at 50% peak torque as determined by the mean and median of percent difference. Equations 1, 2, and 3 underestimated FATI at 50% peak torque, for extension and flexion, respectively, by (1) −11.6% and −12.1%, (2) −23.5 and −28.6%, and (3) −13.9 and −15.1%. Although, the FATI at 30 s underestimates 50% peak torque, the estimation is small, and so FATI at 30 s can be used to replace FATI at 50% peak torque using Approach 1.

**Table 3:** Errors (30 s minus 50%) when FATI at 30 s is used as a replacement for 50% peak torque for the extension fatigability test.

**Equation**	***N***	***$\bar{x}$***	**SD**	**Median**	**Min**	**Max**	**SQRT**	***$\bar{x}$* of abs**	**95% CI (LL)**	**95% CI (UL)**
**Extension**										
1	38	−2.46	2.66	−2.35	−8.50	2.56	3.59	2.90	−7.84	2.92
2	38	−3.59	3.35	−3.19	−10.9	2.39	4.89	3.99	−10.4	3.20
3	38	−3.47	3.13	−3.05	−10.3	2.05	4.64	3.76	−9.81	2.88

*SD, standard deviation; max, Maximum; Min, Minimum; Abs, Absolute; CI, confidence interval; LL, UL, Lower and Upper Limit; SQRT, square-root of the average of errors squared*.

**Table 4:** Errors (30 s minus 50%) when FATI at 30 s is used as a replacement for 50% peak torque for the flexion fatigability test.

**Equation**	**N**	***$\bar{x}$***	**SD**	**Median**	**Min**	**Max**	**SQRT**	***$\bar{x}$* of abs**	**95% CI (LL)**	**95% CI (UL)**
**Flexion**										
1	29	−3.18	2.43	−2.90	−8.80	4.20	3.98	3.47	−8.16	1.81
2	29	−3.89	3.04	−3.55	−11.3	5.43	4.90	4.27	−10.1	2.32
3	29	−3.70	2.68	−3.30	−10.7	4.50	4.54	4.01	−9.18	1.79

*SD, standard deviation; max, Maximum; Min, Minimum; Abs, Absolute; LL, UL, Lower and Upper Limit*.

### Approach 2

**Figure 4:**
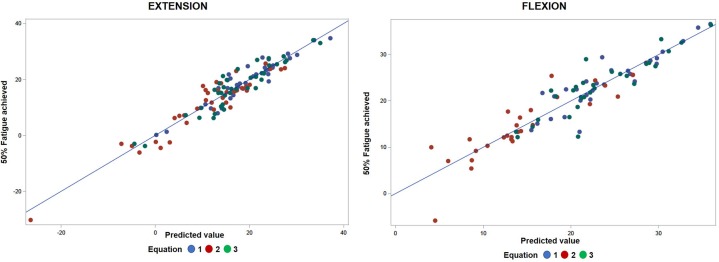
Comparative analyses of predicted FATI at 50% peak torque using Approach 2 (section Approach 2) and actual FATI at 50% peak torque for extension and flexion. Result: observations are spread apart relative to linear line.

Figure 4 shows a smaller estimation bias for the prediction of FATI at 50% peak torque based on FATI at 30 s as compared to Approach 1. The differences between predicted and actual 50% FATI (predicted minus actual 50% FATI) are more balanced around zero than with Approach 1. For this approach, all 3 equations have very small bias (average). However, equation 1 leads to the smallest standard deviation (spread of errors) (2.76), the smallest square-root of uncorrected sum of squares (2.73), and the smallest average of the absolute value of the differences (2.02). These results are shown in Table 5 for extension and similar results were observed for flexion (Table 6). As with approach 1, equation 1 underestimated the FATI at 50% peak torque as determined by the median of % difference, but less than equations 2 and 3 for extension and flexion: (1) 1.0 and 1.2%, (2) −4.2 and 1.9%, and (3) 1.8 and 1.8%.

**Table 5:** Errors (predicted minus actual) from the regression model for the extension fatigability test. Predict 50% peak torque from FATI at 30 s.

**Equation**	***N***	***$\bar{x}$***	**SD**	**Median**	**Min**	**Max**	**SQRT**	***$\bar{x}$* of abs**	**95% CI (LL)**	**95% CI (UL)**
**Extension**										
1	38	0.02	2.76	0.12	−6.20	5.33	2.73	2.02	−5.58	5.62
2	38	0.05	3.46	0.80	−7.56	5.98	3.42	2.87	−6.97	7.07
3	38	0.01	3.18	0.42	−6.82	6.16	3.14	2.45	−6.44	6.46

*SD, standard deviation; max, Maximum; Min, Minimum; Abs, Absolute; LL, UL, Lower and Upper Limit*.

**Table 6:** Errors (predicted minus actual) from the regression model for the flexion fatigability test. Predict 50% peak torque from FATI at 30 s.

**Equation**	***N***	***$\bar{x}$***	**SD**	**Median**	**Min**	**Max**	**SQRT**	***$\bar{x}$* of abs**	**95% CI (LL)**	**95% CI (UL)**
**Flexion**										
1	29	−0.00	2.57	0.28	−5.83	7.69	2.53	1.80	−5.28	5.27
2	29	0.05	3.33	0.38	−7.61	10.4	3.28	2.28	−6.78	6.87
3	29	0.01	2.82	0.40	−7.28	8.53	2.77	1.91	−5.76	5.77

*SD, standard deviation; max, Maximum; Min, Minimum; Abs, Absolute; LL, UL, Lower and Upper Limit*.

### Disease Progression

Given that equation 1, using Approach 2, led to the smallest median error, the smallest square-root of uncorrected sum of squares, and the smallest average of the absolute value of the differences, equation 1 was used to impute values for FATI at 50% peak torque in individuals who did not achieve 50% peak torque during the fatigability test. Imputed and actual values for FATI at 50% peak torque were then used to assess fatigability-based disease progression. No difference was observed in FATI at 50% peak torque between month 0 and month 6 for extension [*N* = 30, *p* = 0.606, mean difference = 0.78, 95% CI = (−2.28, 3.84)] and flexion [*N* = 29, *p* = 0.740, mean difference = 0.45, 95% CI = (−2.30, 3.20)].

## Discussion

Disease severity, and thus physical function, vary among individuals with *RYR1-*RM. For this reason, it can be difficult to obtain consistent test results during fatigability testing in this population for comparison across individuals and/or timepoints. When a muscle can no longer exert a force >50% of the initial peak torque, it is considered to have fatigued (13, 14). Although 60 s fatigability testing inconsistently captured this local muscle fatigue in the quadriceps and hamstrings in this population, the results of this study indicate the FATI from a 30 s test may be leveraged to obtain this information when missing. Objectively identifying differences in local muscle fatigability in this population is a first step toward quantifying underlying mechanisms behind functional limitations often observed among individuals with *RYR1-RM* (9).

Based on the results of this study, local muscle fatigability can be quantified even if fatigue is not reached during a sustained isometric knee fatigability test. Two approaches were compared for the quantification of fatigability in scenarios where objective fatigue was not reached. The prediction-based approach (Approach 2) provided a better estimate of FATI at 50% peak torque, and thus should be used to predict FATI at 50% peak torque when fatigability is not reached.

Within each approach, three equations were assessed to determine the best method for calculating FATI using the output from the fatigability test. Our results suggest equation 1 provided the most accurate representation of fatigability over time because the predicted values were closer to the actual FATI at 50% peak torque when this equation was used. This indicates that there might be important information about fatigability captured during the portion of the test when the subject approaches his or her maximum torque, as equation 1 is the only equation of the three that incorporates this information.

When Approach 2 was used with equation 1 to assess change in local muscle fatigability over a 6-month period in individuals with *RYR1-*RM, no significant change was found. This was expected and supports existing literature, which considers *RYR1-*RM to clinically present as a stable or slowly progressive disease (15, 16).

### Limitations

Unlike Surakka et al., we were not able to determine which equation provided the most accurate FATI at 50% peak torque because we only utilized one test to measure fatigability. Instead, this analysis pointed to the equation that led to the smallest “error” when one imputed the equation's FATI at 50% peak torque based on its FATI at 30 s. However, the present study did determine a method for obtaining information about FATI at 50% when it is not found during fatigability testing.

Another limitation was that the current study only evaluated ambulatory individuals with *RYR1-*RM. While the results of the study may not be directly applicable to the population with *RYR1-*RM who are non-ambulatory, the fatigability testing and statistical methods can be applied to non-ambulatory individuals in the future. This type of analysis may prove particularly useful during intervention studies to quantify small changes in muscle function that may be difficult to capture clinically in this population.

Lastly, reproducibility of this analysis would require MATLAB software and a programmer, which may not be readily available at a clinical center unless research based.

## Conclusion

With either approach, when fatigability was objectively measured in *RYR1-*RM during a sustained knee isometric fatigability test, equation 1 was found be the most accurate at imputing FATI at 50% peak torque based on its FATI at 30 s for both extension and flexion. Both Approach 1 (value-replacement based) and Approach 2 (model-based) showed value in this estimation with the former being easier to implement and the latter leading to more accurate imputed values. Furthermore, no difference in local muscle fatigue was observed at 50% peak torque between month 0 and month 6 for either knee extension or flexion thereby demonstrating fatigability is stable over 6 months in the *RYR1-*RM population.

Future work should build upon this foundation to explore if Approach 2, which was found to be most accurate in this analysis and also best represents muscle fatigability as measured by motor-unit activity (17). However, during the study, two participants experienced knee pain after performing this testing. After this occurred, the protocol was amended to exclude individuals with a history of knee injury from performing this procedure, and this issue did not recur. Given leg muscle weakness in this population, especially of the thigh muscles, we recommend exclusion of *RYR1*-RM affected individuals with a history knee pain from knee isometric testing as a precaution. Additionally, future studies should include both ambulatory and non-ambulatory individuals with *RYR1-*RM to better understand specific adjustments, if any, needed to best utilize this method in each sub-population.

## Data Availability Statement

The datasets generated for this study will not be made publicly available. The datasets are stored in a secure database. They will be made available be available upon request. Point of contact is KM.

## Ethics Statement

The studies involving human participants were reviewed and approved by Institutional Review Board (IRB) at NIH. Written informed consent to participate in this study was provided by the participants' legal guardian/next of kin.

## Author Contributions

JW collected and managed the data. JR is responsible for equations and writing the program to yield final results from raw data. PW is responsible for all statistics. RV and MW assisted with data collection. IC was responsible for patient recruitment and scheduling. MS assisted with data management. MJ assisted with data collection and is the head of research and develop in the Rehabilitation Medicine Department. KM is the PI of the lab.

### Conflict of Interest

The authors declare that the research was conducted in the absence of any commercial or financial relationships that could be construed as a potential conflict of interest.

## Supplementary Material

Supplementary Figure 1 Sample MVIC curve where the calculation of FATI_1_ would be B/(A+B+C) where B is the dark shaded area under the MVIC curve from time 0 to the end of the test, and A and C are the light shaded regions above the MVIC curve representing the areas that would have been included in the test had the participant instantaneously reached their maximum force and not fatigued at all during the test. A smaller value for FATI_1_ indicates a greater amount of fatigability. Adapted from Surakka et al. (12).

Supplementary Figure 2 Sample MVIC curve where the calculation of FATI_2_ would be D/(D+E) where D is the dark shaded area under the MVIC curve from 5 s to the end of the test, and E is the light shaded area above the MVIC curve after the first 5 s of the test representing the area that would have been included in the test had the participant not fatigued at all after the first 5 s. A smaller value for FATI_2_ indicates a greater amount of fatigability after the first 5 s of the test. Adapted from Surakka et al. (12).

Supplementary Figure 3 Sample MVIC curve where the calculation of FATI_3_ would be F/(F+G) where F is the dark shaded area under the MVIC curve from the timepoint of maximum force to the end of the test, and G is the light shaded area above the MVIC curve after the timepoint of maximum force representing the area that would have been included in the test had the participant not fatigued at all after reaching their maximum force. A smaller value for FATI_3_ indicates a greater amount of fatigability after the time the participant reached their maximum force. Adapted from Surakka et al. (12).
