# Supplementary figures and images for: Use of Fatigue Index as a Measure of Local Muscle Fatigability in Ryanodine Receptor Isoform-1-Related Myopathies
Source: Front Neurol. 2019 Dec 10;10:1234. doi: 10.3389/fneur.2019.01234 (PMC6914942; doi:10.3389/fneur.2019.01234)

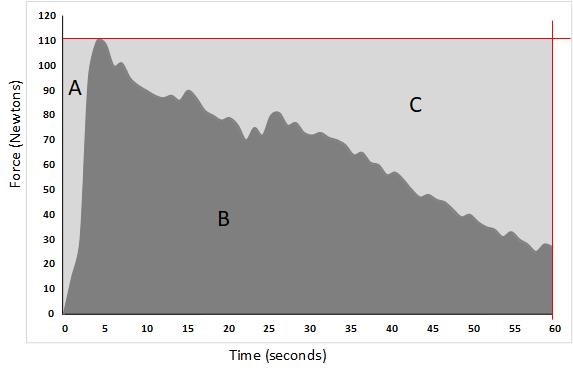

Supplement: Supplementary Figure 1 — Sample MVIC curve where the calculation of FATI1 would be B/(A+B+C) where B is the dark shaded area under the MVIC curve from time 0 to the end of the test, and A and C are the light shaded regions above the MVIC curve representing the areas that would have been included in the test had the participant instantaneously reached their maximum force and not fatigued at all during the test. A smaller value for FATI1 indicates a greater amount of fatigability. Adapted from Surakka et al. (12). [file Image_1.jpeg]

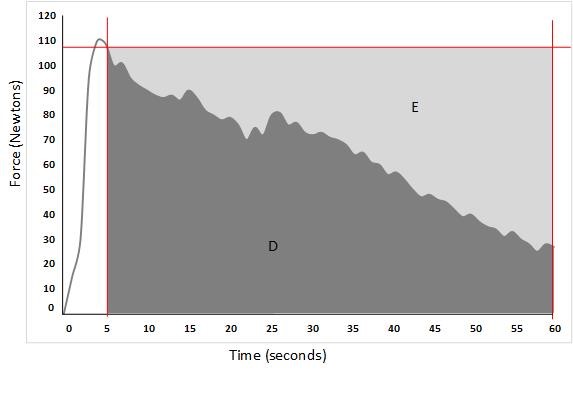

Supplement: Supplementary Figure 2 — Sample MVIC curve where the calculation of FATI2 would be D/(D+E) where D is the dark shaded area under the MVIC curve from 5 s to the end of the test, and E is the light shaded area above the MVIC curve after the first 5 s of the test representing the area that would have been included in the test had the participant not fatigued at all after the first 5 s. A smaller value for FATI2 indicates a greater amount of fatigability after the first 5 s of the test. Adapted from Surakka et al. (12). [file Image_2.jpeg]

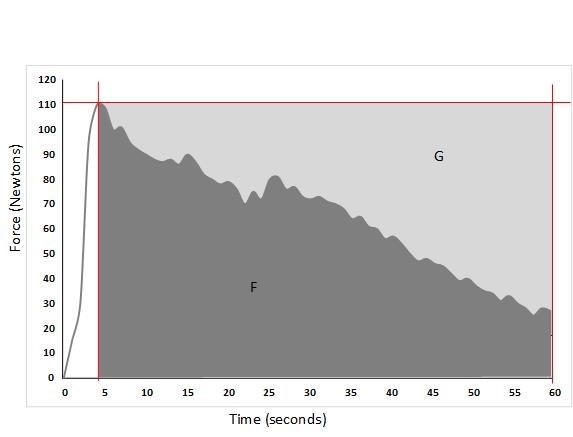

Supplement: Supplementary Figure 3 — Sample MVIC curve where the calculation of FATI3 would be F/(F+G) where F is the dark shaded area under the MVIC curve from the timepoint of maximum force to the end of the test, and G is the light shaded area above the MVIC curve after the timepoint of maximum force representing the area that would have been included in the test had the participant not fatigued at all after reaching their maximum force. A smaller value for FATI3 indicates a greater amount of fatigability after the time the participant reached their maximum force. Adapted from Surakka et al. (12). [file Image_3.jpeg]
